# Experimental datasets of networks of nonlinear oscillators: Structure and dynamics during the path to synchronization

**DOI:** 10.1016/j.dib.2019.105012

**Published:** 2019-12-18

**Authors:** V.P. Vera-Ávila, Ricardo Sevilla-Escoboza, A.A. Lozano-Sánchez, R.R. Rivera-Durón, Javier M. Buldú

**Affiliations:** a Centro Universitario de los Lagos, Universidad de Guadalajara, Jalisco, 47460, Mexico; b Institute of Unmanned System and Center for Optical Imagery Analysis and Learning (OPTIMAL), Northwestern Polytechnical University, Xi'an, 710072, China; c Complex System Group & GISC, Universidad Rey Juan Carlos, 28933, Móstoles, Madrid, Spain; d Center for Biomedical Technology, UPM, 28223, Pozuelo de Alarcón, Madrid, Spain

**Keywords:** Synchronization, Complex networks, Nonlinear dynamics, Chaos, Electronic circuits

## Abstract

The analysis of the interplay between structural and functional networks require experiments where both the specific structure of the connections between nodes and the time series of the underlying dynamical units are known at the same time. However, real datasets typically contain only one of the two ways (structural or functional) a network can be observed. Here, we provide experimental recordings of the dynamics of 28 nonlinear electronic circuits coupled in 20 different network configurations. For each network, we modify the coupling strength between circuits, going from an incoherent state of the system to a complete synchronization scenario. Time series containing 30000 points are recorded using a data-acquisition card capturing the analogic output of each circuit. The experiment is repeated three times for each network structure allowing to track the path to the synchronized state both at the level of the nodes (with its direct neighbours) and at the whole network. These datasets can be useful to test new metrics to evaluate the coordination between dynamical systems and to investigate to what extent the coupling strength is related to the correlation between functional and structural networks.

**Table:** Specifications Table

Subject	Physics
Specific subject area	Nonlinear dynamics, complex networks, synchronization.
Type of data	- Tables - Graphs - Figures - Times series in text file
How data were acquired	- We used a Data Acquisition Card (DAQ), NI USB-6363 to acquire the voltage of N = 28 nonlinear electronic oscillators connected through a complex network. - 20 different network structures were implemented. - For each structure, the time series (30000 points) corresponding to 100 different coupling strengths are recorded. - The full experiment is repeated three times for each network structure
Data format	- Raw Data. - Time series are given in a plain text file.
Parameters for data collection	- Sampling frequency of 30KS/s - Filtered using a Butterworth order 3 low-pass filter with a cut-off frequency of 1500 Hz. - 30000 points for each time series - Resolution of 16 bits - Range of the ADC: Form −5 V–5 V
Description of data collection	Datasets consists of the structure and dynamics of electronic arrays of *N* = *28* Rössler circuits operating at the chaotic regime. The configuration of he coupling variables make the circuits to be class II system in the classification given by the Master Stability Function. Using a multichannel DAQ, the voltage corresponding to one of the variables of each Rössler oscillator was acquired using the analogue ports (AI-0, AI-1, …., AI-27). An in-house electronic coupler was the responsible of defining the structure and strength of the coupling between the oscillators. 20 different network structures were implemented and recorded. A digital potentiometer was used to change the coupling strength between the nodes, which was controlled by means of the digital ports (P0.0 P0.1) of the DAQ card. In this way, we were able to turn the dynamics of the network from an incoherent behaviour (low coupling strength) to a fully synchronized state (high coupling strength).
Data source location	Institution: Centro Universitario de los Lagos (Universidad de Guadalajara) City/Town/Region: Lagos de Moreno, Jalisco. Country: Mexico
**Data accessibility**	With the article Repository name: [Zenodo] Data identification number: [10.5281/zenodo.3521009] Direct URL to data: https://doi.org/10.5281/zenodo.3521009

**Table:** 

**Value of the Data** • We provide the time series of a *N* = *28* networked system in its path to synchronization for 20 different network structures. Datasets allow investigating the interplay between the dynamics of a set of nonlinear dynamical systems with the specific underlying structure and the coupling needed by the network to synchronize. • Datasets can be used to develop new metrics to evaluate synchronization between nonlinear systems. However, the main merit of the datasets is having at the same time the structure and dynamics of 20 different networks. This is not so common since, in most real cases, we have datasets concerning only the structure of the networks (e.g., road networks, power grids, cortical networks, …) or only the dynamics (e.g., electroencephalography, functional magnetic resonance). Therefore, studies about the interplay between structural and functional networks could benefit from the current datasets. • It is one of the very few cases where the structure and dynamics of a networked system is known (with precision) at the same time. In addition, the existence of the intrinsic noise and tolerance of the electronic components can be used as an evidence of the robustness of the eventual analysis.

## 1 Data

**Fig. 1:**
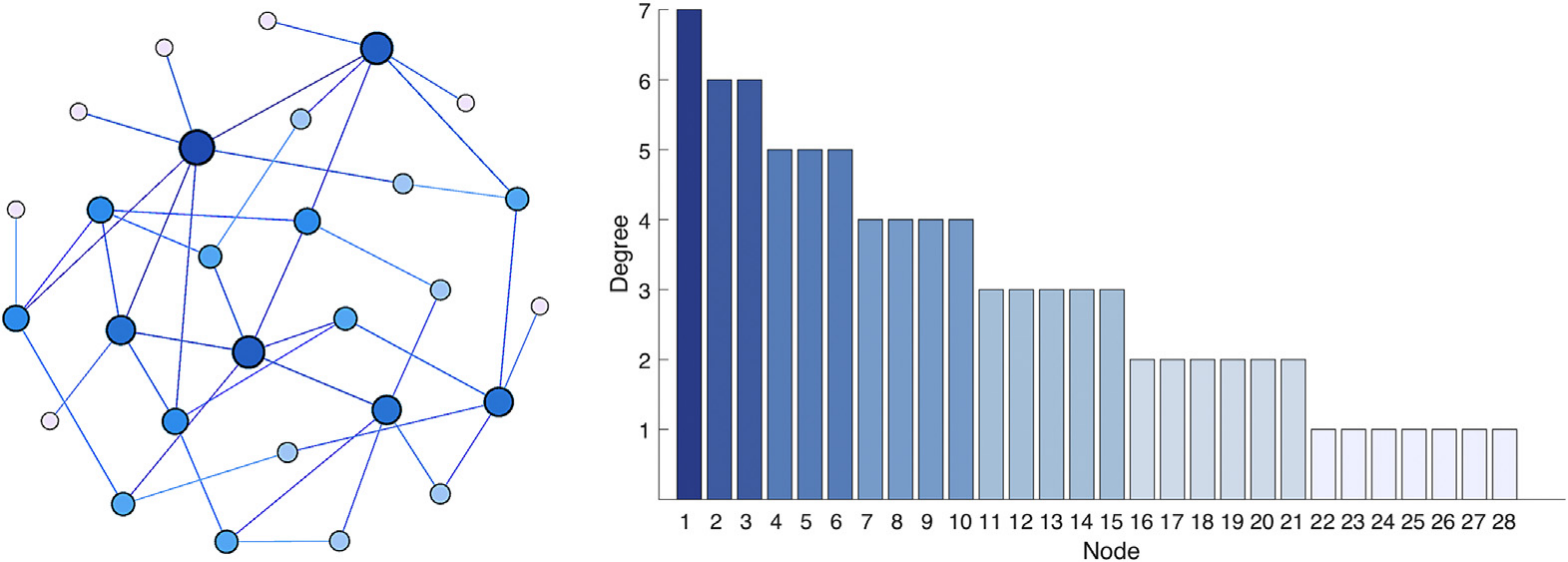
Schematic representation of the structural networks. (Left) Example of one network structure used for the experiment; the size of the nodes is proportional to their degree (number of neighbours). (Right) Degree distribution of the nodes, which was maintained for the 20 network structures.

**Fig. 2:**
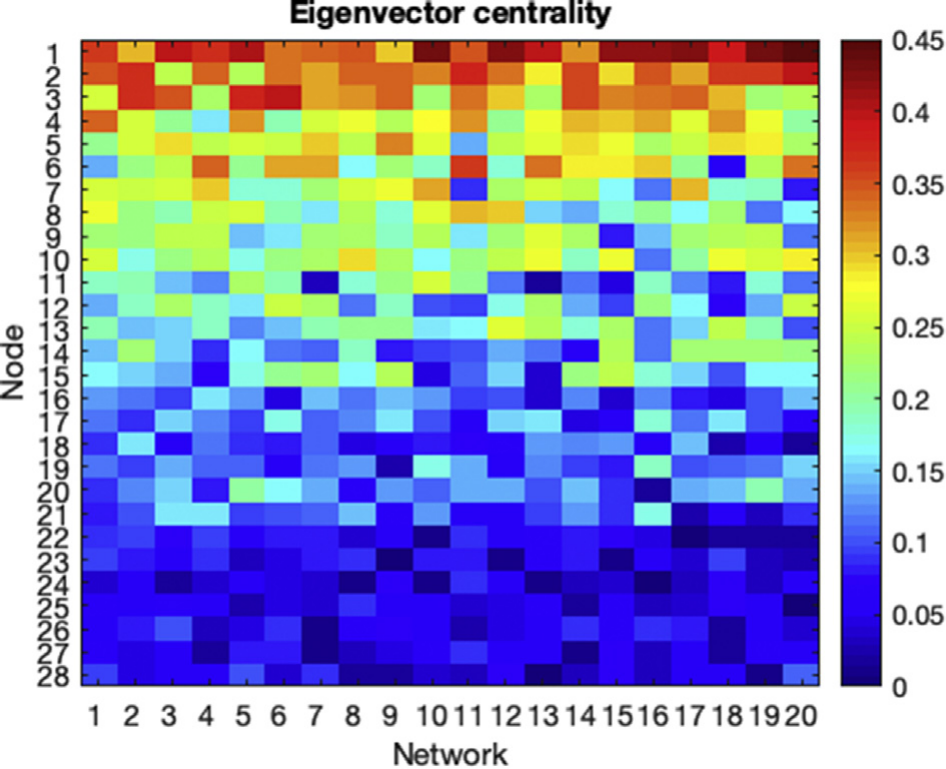
Eigenvector centrality. Node centrality for each of the 20 network structures. Warm colors correspond to nodes with high centrality.

**Fig. 3:**
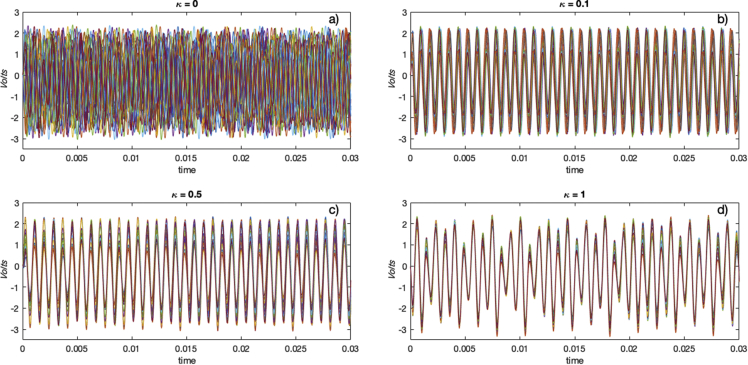
Time series of the second variable *v*_*2*_ (see Eqs (1), (2), (3)) of the oscillators of Network 1 at different coupling values for: a) File ST_1_1 (κ=0), b) File ST_10_1 (κ=0.1), c) File ST_50_1 (κ=0.5), and d) File ST_100_1 (κ=1).

**Fig. 4:**
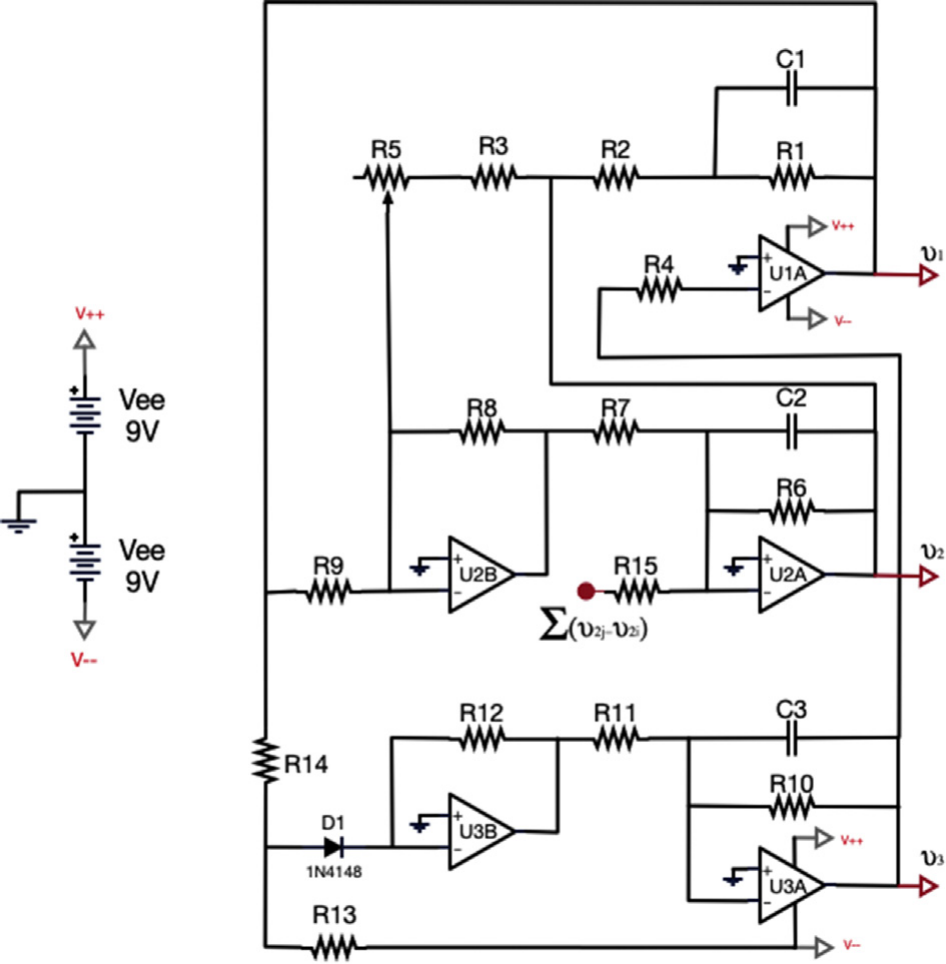
Electronic representation of the Rössler-like system. See the specific values of the resistances and capacitors at Table 1.

**Fig. 5:**
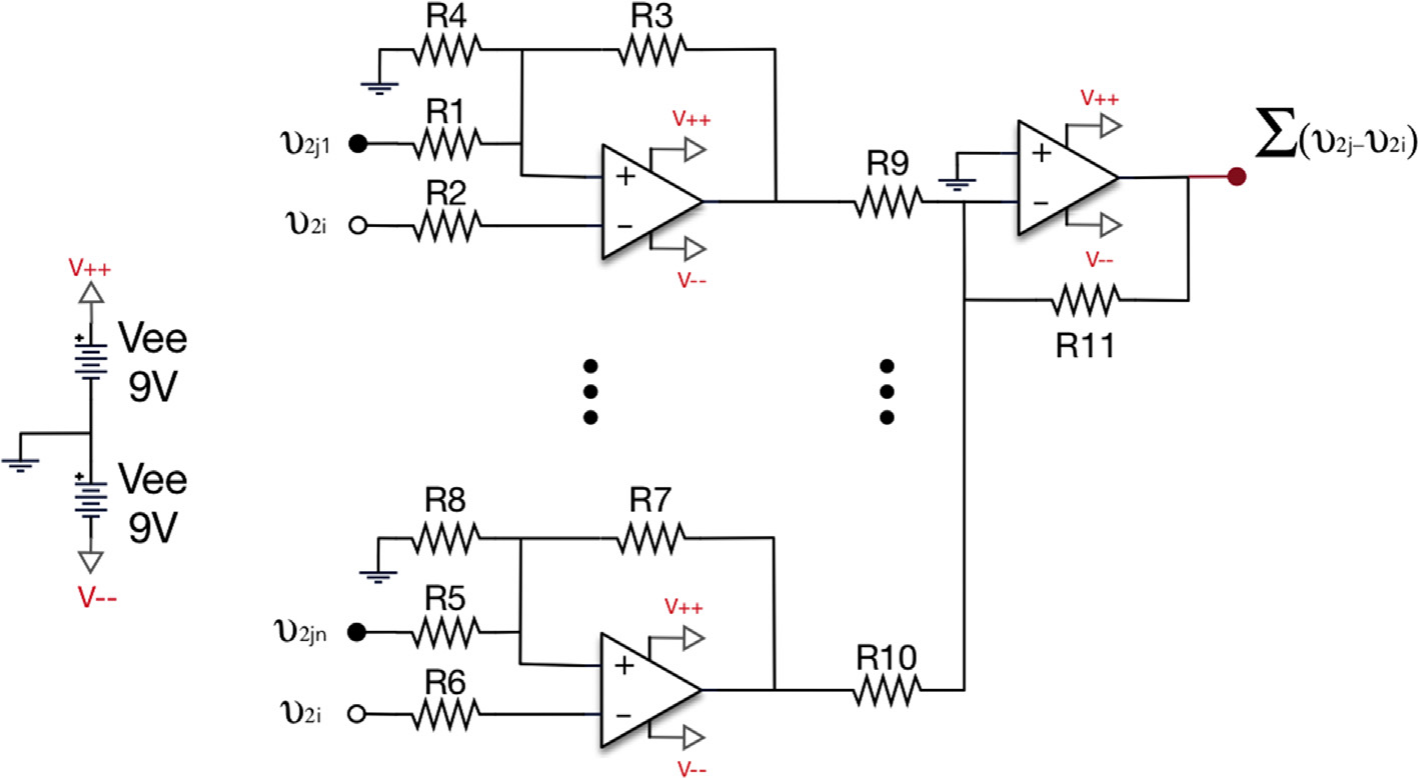
Electronic representation of the coupler circuit responsible of the diffusive coupling between an electronic oscillator and its neighbours. We use an operational amplifier (Op-Amp) for the output of oscillator i and each of its j neighbours. Finally, we sum the input of all incoming neighbours. In the coupler circuit, all resistances are set to 1 kΩ*.*

**Fig. 6:**
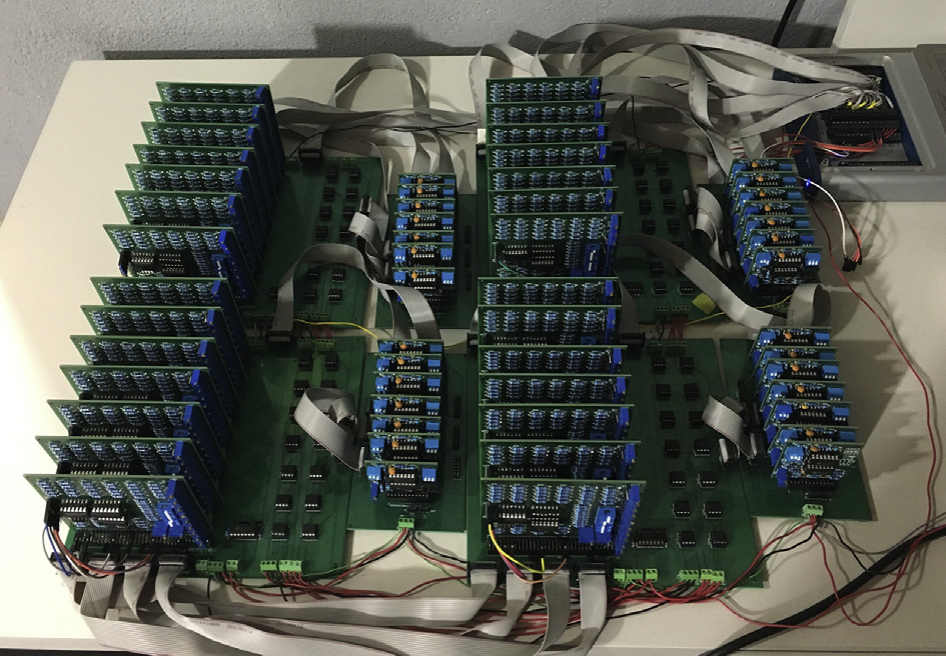
Photograph of the experimental array using *28* nonlinear oscillators and their corresponding electronic couplers. The voltage of each oscillator was acquired by a DAQ card (*NI USB 6363*), shown at the top right corner of the photograph.

**Fig. 7:**
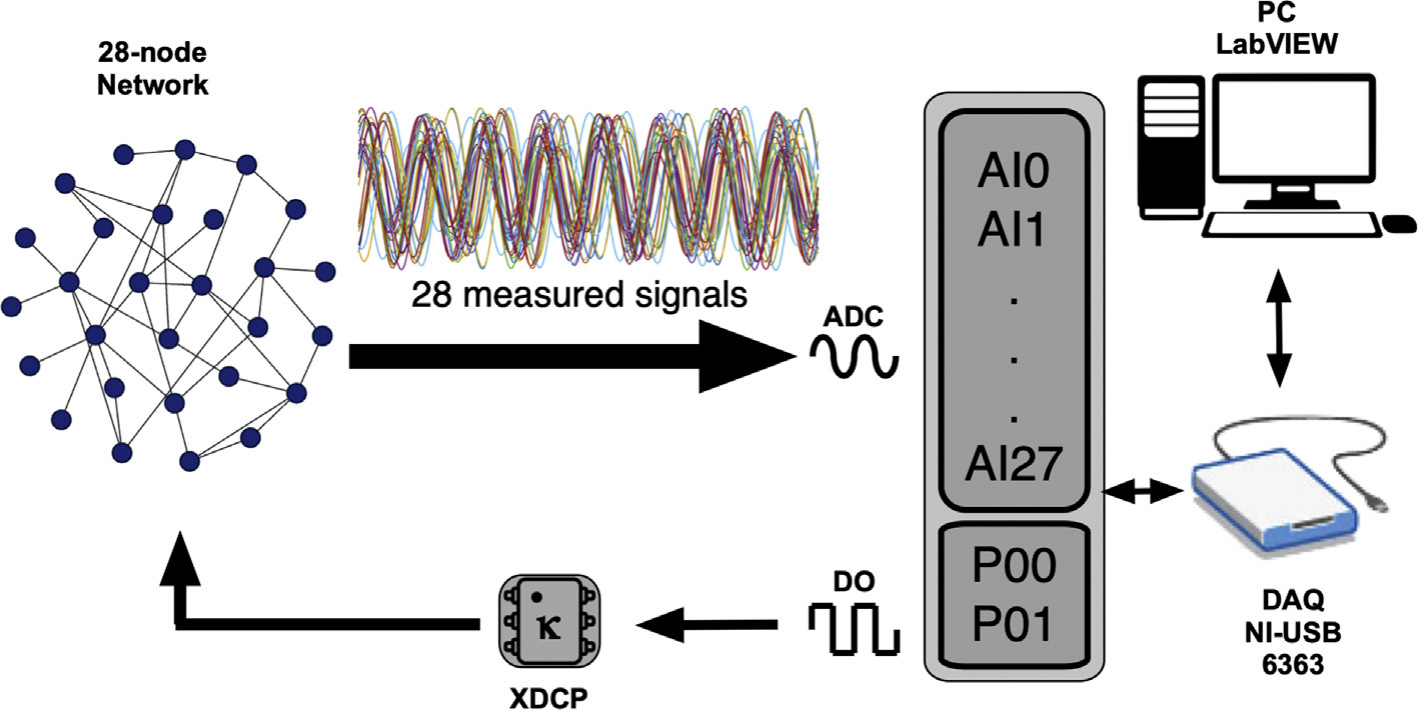
Schematic representation of the experimental setup of a network of 28 nonlinear electronic circuits. The coupling strength was adjusted by means of digital potentiometers XDCP (model X9C103). Coupling resistances were controlled through digital pulses sent by a DAQ (NI USB 6363). All signals were acquired using analogic ports (AI-0 to AI-27). The experiment was controlled using the LabVIEW software.

The datasets contain the structure of 20 networks composed of 28 Rössler electronic oscillators, with each network having a different structure. The number of oscillators is limited by the analogue ports of the data acquisition card (DAQ), however, it is fully scalable as long as a DAQ with higher number of analogue ports is used. We also include the dynamics of the 28 nodes for 100 different values of the coupling strength, which allows tracking the path to synchronization in all structural networks. Fig. 1 describes the network structure and the degree distribution for all the experiments, the latter being maintained for all network structures. Fig. 2 shows the eigenvector centrality of each node for all network structures. Fig. 3 describes the evolution of the time series when the coupling strength between nodes is increased. Fig. 4 shows the Rössler oscillators placed at the nodes of the networks. Fig. 5 describes the coupling circuit between nodes. Fig. 6, Fig. 7 show a photograph of the experimental array and the corresponding schematic representation.

## 2 Experimental design, materials, and methods

We provide the times series of *N* = *28* Rössler electronic oscillators for *20* different network configurations (compressed file with tags from R1 to R20). For each network structure, we recorded the times series for *101* different coupling strengths between oscillators. Each one of the *101* corresponding files is labelled as *ST_X_Y.dat* where *X* is a value between *X* = *0 and X* = 100 that corresponds to the minimum *(*κ = *0)* and maximum coupling strength (κ = *1)*, respectively. The value of *Y* indicates the number of repetition, which can be *1, 2* of *3* (i.e., we repeated the same experiment three times). Data files contain the second variable of the *28* nodes (see variable $v_{2}$ of (1), (2), (3)) arranged in columns with a length of *30000* points. In a second file named *Structure.zip*, all network structures are given, each file having a name *Net_R.dat*, where *R* = *1, 2, …, 20*. The degree of each node (i.e., number of output connections) is the same for all network configurations (see Fig. 1, right plot). However, the specific neighbours of each node are re-arranged randomly at each network structure.

### 2.1 Network properties

The structure of the *20* different network configurations is random. First, we assigned the degree of the nodes in order to have a high heterogeneity, i.e., we fixed the degree distribution. The reason is that we wanted to have hubs and also nodes with a low degree, with the aim of promoting further works relating the degree of the nodes with their synchronization properties. Next, for each structural network, we re-arranged the connection between nodes maintaining their degree. In this way, we generated a group of datasets were the structure of the networks, and not the degree distribution, induced changes in the dynamics. As a consequence, the centrality of the nodes (i.e., its importance in the network) changed from network to network. To show this point, we computed the *eigenvector centrality* [4] of each node. In Fig. 2 we can observe the change of the node's centrality for each network structure.

In Fig. 3 there is an example of the transition to synchronization, we can see the time series of the second variable of the electronic oscillator. In Fig. 3(a) we can see how when the coupling is zero (κ=0), the oscillators evolve independently. However, when the coupling increases (κ=0.1 in b and κ = 0.5 in c); oscillators begin to evolve together, until they reach complete synchronization (κ=1 in d). The value of the coupling strength κ is proportional to the incoming impedance to each oscillator of the network [see Eq. (2)].

### 2.2 The dynamical system

Each node of the network contains a Rössler-like nonlinear oscillator. The detailed components and connections are shown in Fig. 4. The dynamics of an oscillator i is given by the following equations [2,3]:

(1) $${\dot{v}}_{1i}=-\frac{1}{R_{1}C_{1}}\left(v_{1i}+\frac{R_{1}}{R_{2}}v_{2i}+\frac{R_{1}}{R_{4}}v_{3i}\right)$$

(2) $${\dot{v}}_{2i}=-\frac{1}{R_{6}C_{2}}\left(\frac{R_{6}R_{8}}{R_{9}R_{7}}v_{1i}+\left[1-\frac{R_{6}R_{8}}{R_{C}R_{7}}\right]v_{2i}-\kappa \frac{R_{6}}{R_{15}}\sum_{j=1}^{N}A_{ij}\left(v_{2j}-v_{2i}\right)\right)$$

(3) $${\dot{v}}_{3i}=-\frac{1}{R_{10}C_{3}}\left(-\frac{R_{10}}{R_{11}}G\left(v_{1i}\right)+v_{3i}\right)$$

where $v_{1}$, $v_{2}$ and $v_{3}$ correspond to the three voltages describing the dynamical state of each electronic oscillator, is the adjacency matrix containing the specific structure of the network ($a_{ij}$ = *1* if circuit i is connected to circuit j, and *0* otherwise), $R_{i}$ are resistances, $C_{i}$ are capacitors (see Table 1) and $G_{v_{1i}}$ is a piecewise nonlinear function given by:

$$G_{v_{1i}}=\{\begin{array}{c} \;\;\;\;\;\;\;\;\;\;\;\;\;0,\;\;\;v_{1}\leq V_{d}+V_{d}\frac{R_{14}}{R_{13}}+V_{ee}\frac{R_{14}}{R_{13}}\\ \frac{R_{12}}{R_{14}}v_{1i}-V_{ee}\frac{R_{12}}{R_{13}}-Vd\left(\frac{R_{12}}{R_{13}}+\frac{R_{12}}{R_{14}}\right),\;\;\;v_{1}>V_{d}+V_{d}\frac{R_{14}}{R_{13}}+V_{ee}\frac{R_{14}}{R_{13}} \end{array}$$

**Table 1:** Values of the electronic components of the Rössler-like circuits, which have a chaotic output.

C_1_ = 1nF	C_2_ = 1nF	C_3_ = 1nF	κ = [0–1]
R_1_ = 2MΩ	R_2_ = 200kΩ	R_3_ = 10kΩ	R_4_ = 100kΩ
R_5_ = 50kΩ	R_6_ = 5MΩ	R_7_ = 100kΩ	R_8_ = 10kΩ
R_9_ = 10kΩ	R_10_ = 100kΩ	R_11_ = 100kΩ	R_12_ = 150kΩ
R_13_ = 68kΩ	R_14_ = 10kΩ	R_15_ = 500kΩ	R_C_ = 58kΩ
V_d_ = 0.7	V_ee_ = 9		

The output voltage $v_{2}$ of each nonlinear oscillator is sent to an electronic coupler to introduce the diffusive coupling $\left(v_{2_{j}}-v_{2_{i}}\right)$ between oscillator i and each of itsj neighbours (see Fig. 5).

In the case of the Rössler oscillator the coupling through variable $v_{2}$ ensures that the system is class II, following the classification of the *Master Stability Function*, and therefore the ensemble will be able of synchronizing above a threshold value of κ for any topological configuration [1].

### 2.3 The experimental setup

The coupling strength between the nonlinear electronic circuits was controlled by means of digital potentiometers (model X9C103), which acted as voltage divisors with a maximum resistance of 10 kΩ corresponding to a zero coupling. These potentiometers were controlled through the digital ports (P0.0, P0.1) of a DAQ card. First, we set the coupling value of all circuits to zero and then, after a transitory of 500 ms, we recorded the time series of each network: All variables $v_{2_{i}}$ of each oscillator i were acquired by the DAQ card through the analogue ports (AI0, AI1, …, AI27) and saved into the computer. Next, the coupling strength between all nodes was increased one step (0.01) by means of digital pulses that were sent to the potentiometers, decreasing the coupling resistance *100Ω* each step, until the maximum value of κ was reached (i.e.*,* when digital potentiometers are set to *0 Ω*). All the experiment was controlled from a computer using the LabVIEW software (see Fig. 6, Fig. 7).
