# Supplementary figures and images for: Experimental datasets of networks of nonlinear oscillators: Structure and dynamics during the path to synchronization
Source: Data Brief. 2019 Dec 18;28:105012. doi: 10.1016/j.dib.2019.105012 (PMC6961064; doi:10.1016/j.dib.2019.105012)

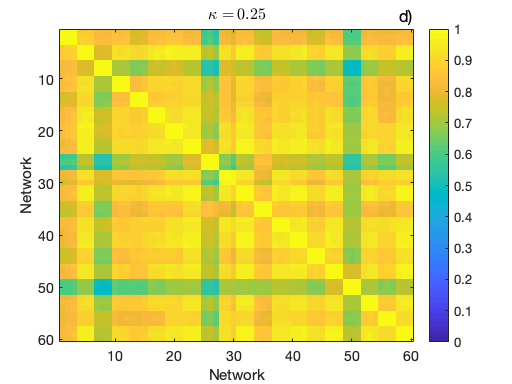

Supplement: Multimedia component 1 [file mmc1.zip › Corr_k025.png]

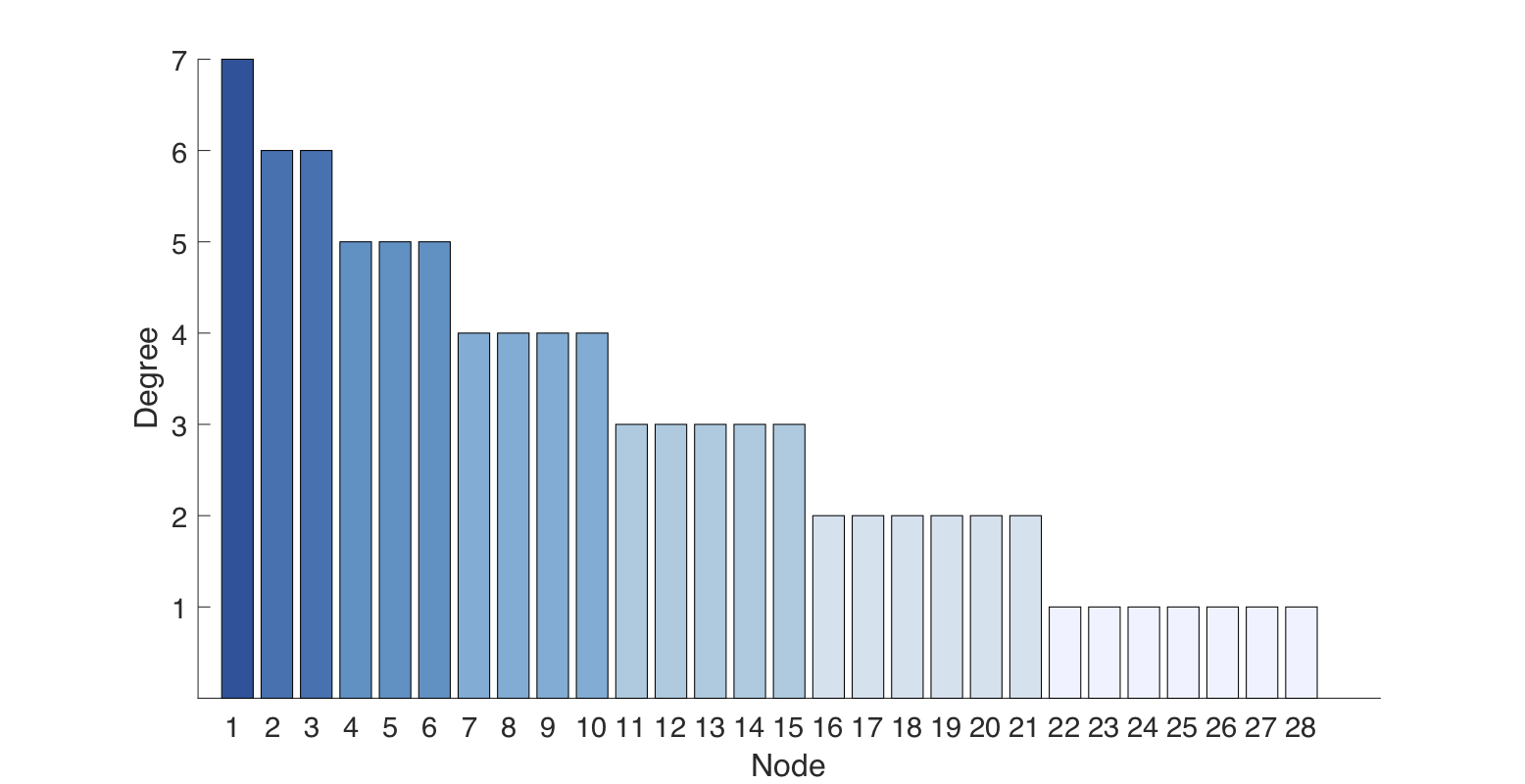

Supplement: Multimedia component 1 [file mmc1.zip › Degree.png]

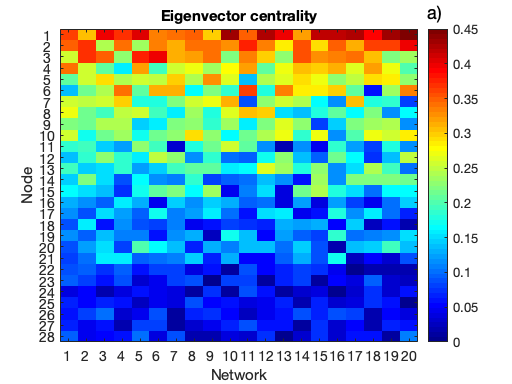

Supplement: Multimedia component 1 [file mmc1.zip › eig_centrality.png]

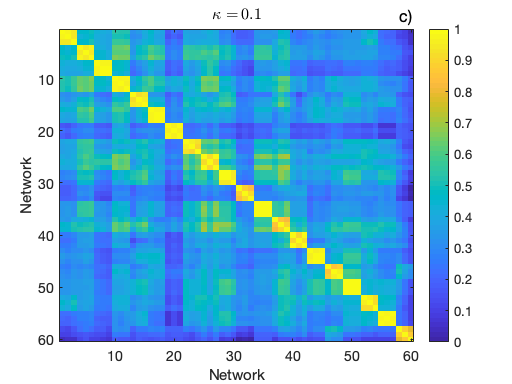

Supplement: Multimedia component 1 [file mmc1.zip › Corr_k01.png]

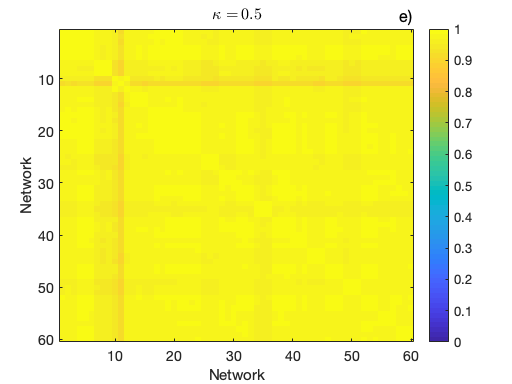

Supplement: Multimedia component 1 [file mmc1.zip › Corr_k05.png]

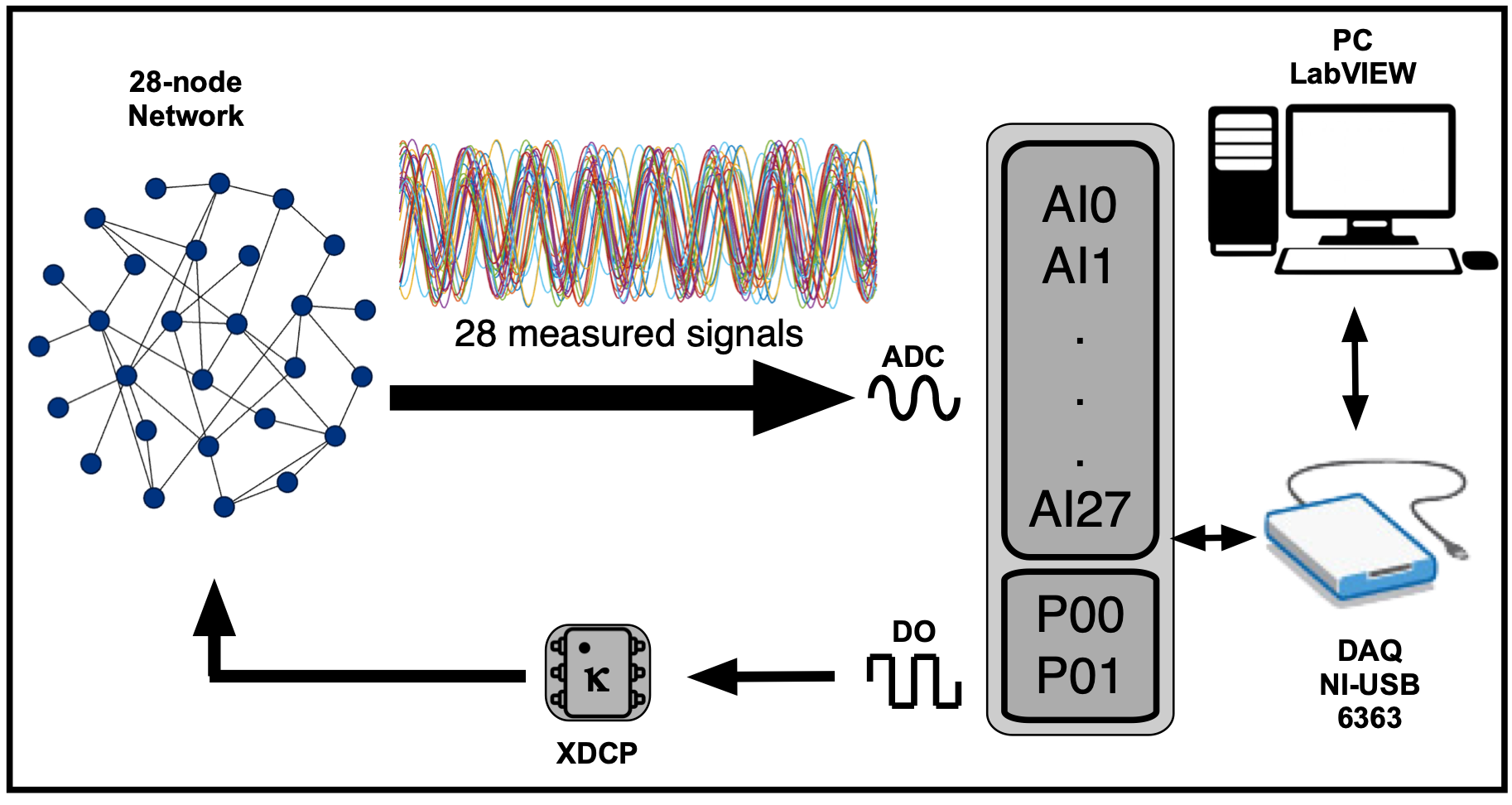

Supplement: Multimedia component 1 [file mmc1.zip › Setup_experimento.png]

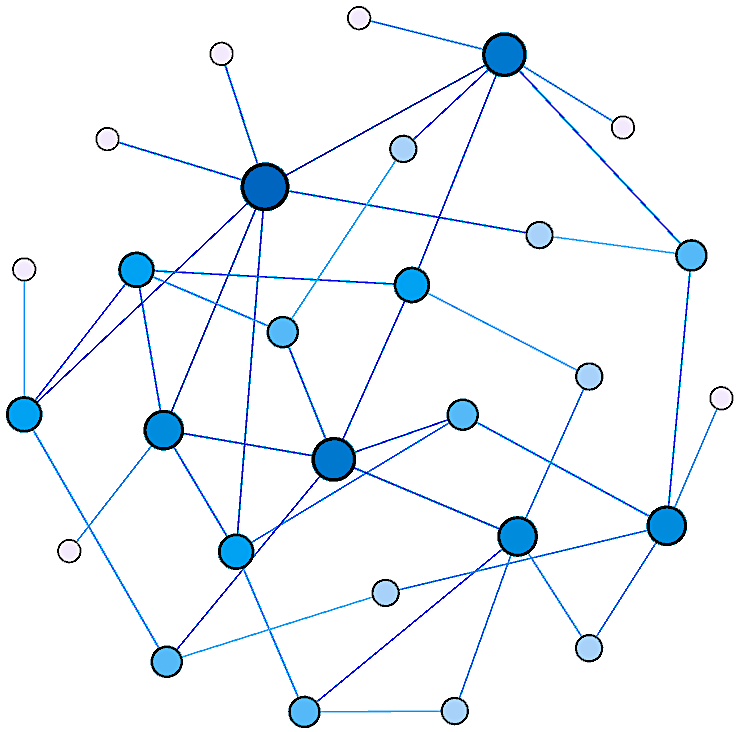

Supplement: Multimedia component 1 [file mmc1.zip › Graph_DB.png]

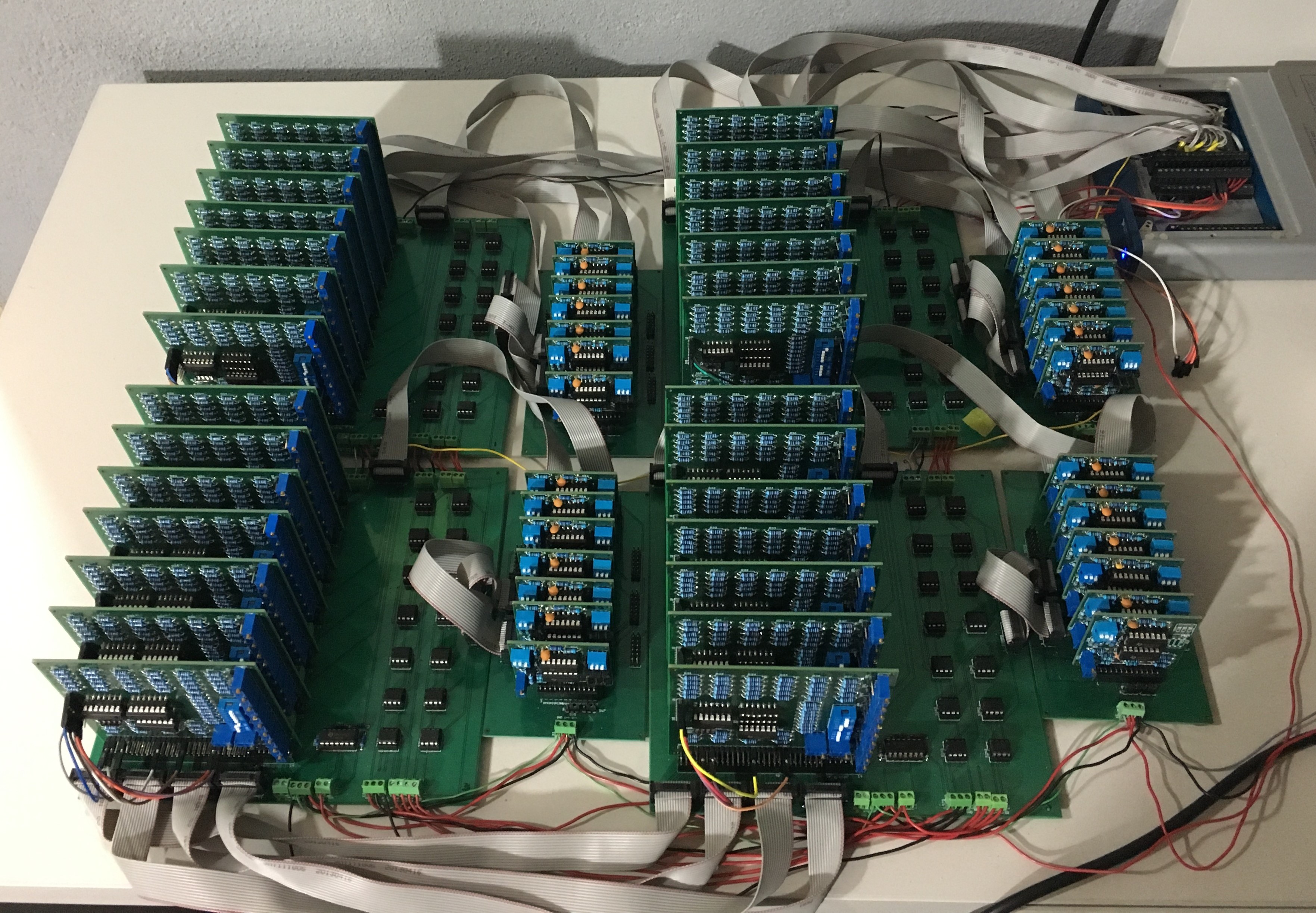

Supplement: Multimedia component 1 [file mmc1.zip › arreglo.JPG]

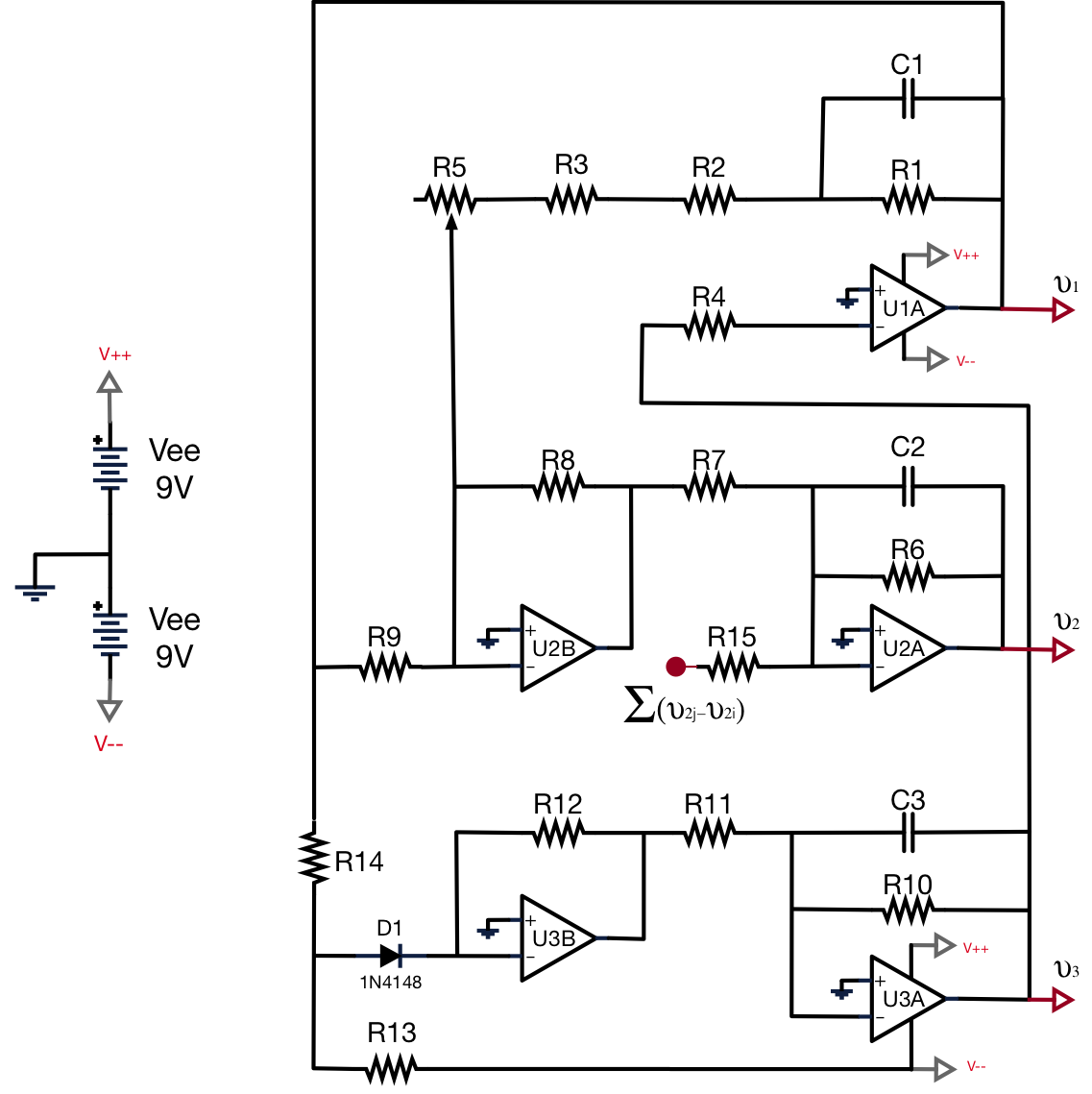

Supplement: Multimedia component 1 [file mmc1.zip › Ross_circuit.png]

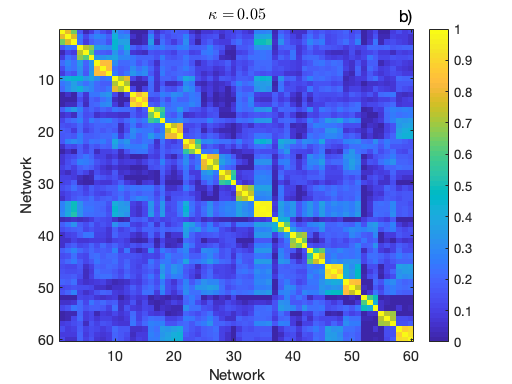

Supplement: Multimedia component 1 [file mmc1.zip › Corr_s005.png]

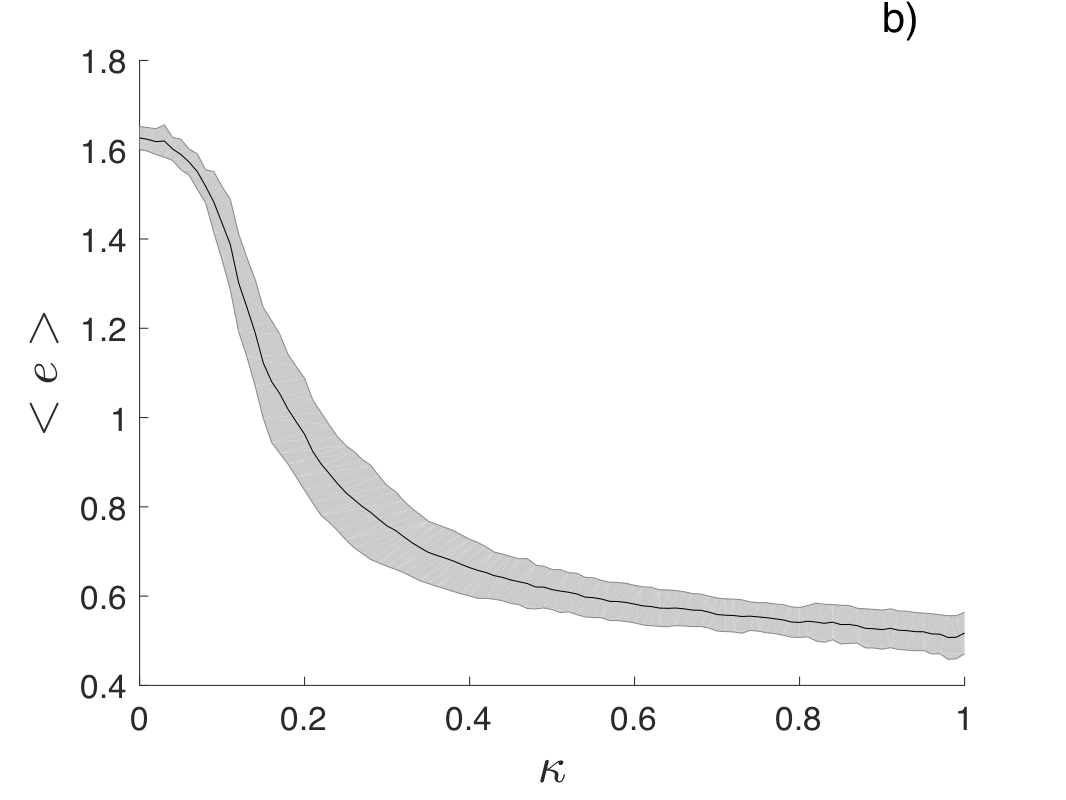

Supplement: Multimedia component 1 [file mmc1.zip › Error_global.png]

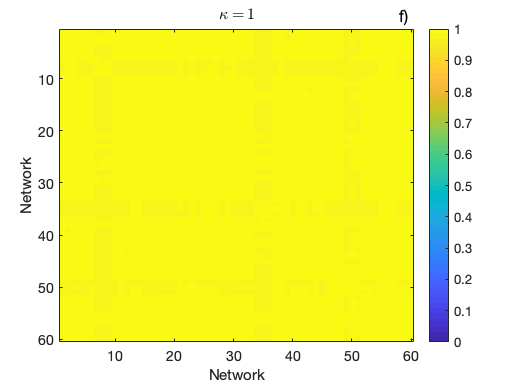

Supplement: Multimedia component 1 [file mmc1.zip › Corr_k1.png]

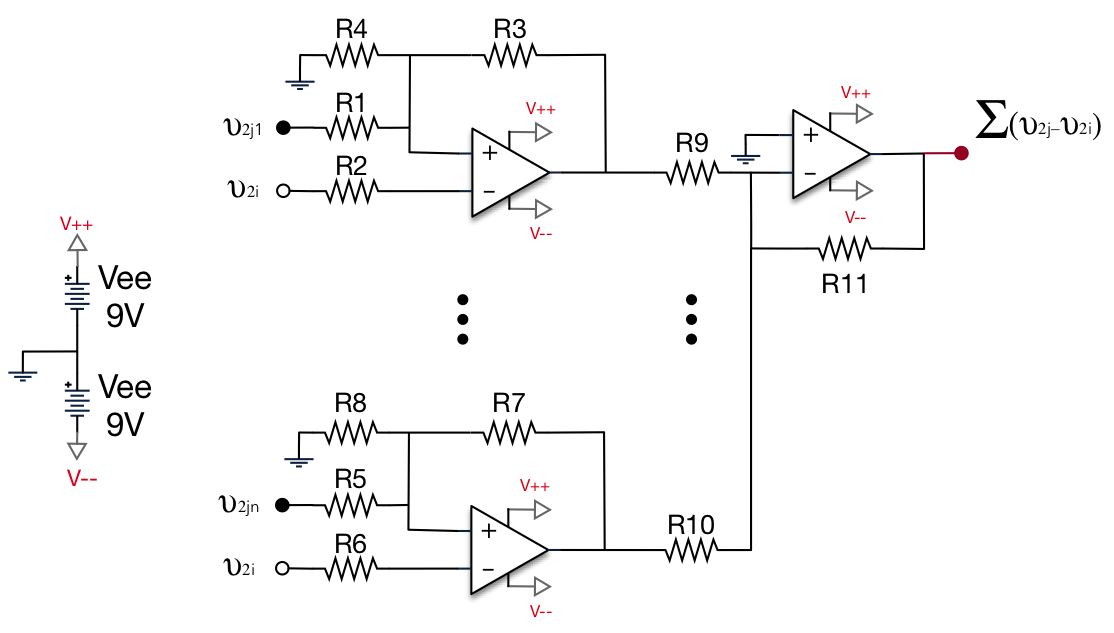

Supplement: Multimedia component 1 [file mmc1.zip › Acoplador_omni.png]

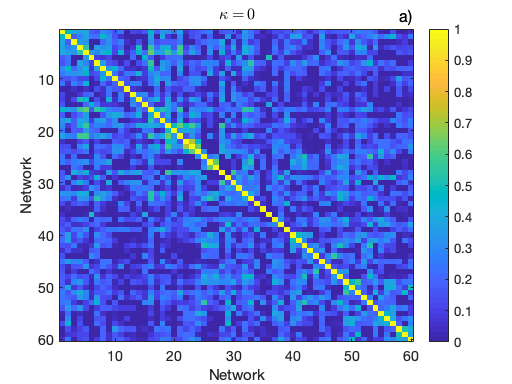

Supplement: Multimedia component 1 [file mmc1.zip › Corr_k0.png]
